# Improving the accuracy of protein secondary structure prediction using structural alignment

**DOI:** 10.1186/1471-2105-7-301

**Published:** 2006-06-14

**Authors:** Scott Montgomerie, Shan Sundararaj, Warren J Gallin, David S Wishart

**Affiliations:** 1 Department of Computing Science, University of Alberta, Edmonton, AB, T6G 2E8, Canada; 2 Department of Biological Sciences, University of Alberta, Edmonton, AB, T6G 2E9, Canada

## Abstract

**Background:**

The accuracy of protein secondary structure prediction has steadily improved over the past 30 years. Now many secondary structure prediction methods routinely achieve an accuracy (Q3) of about 75%. We believe this accuracy could be further improved by including structure (as opposed to sequence) database comparisons as part of the prediction process. Indeed, given the large size of the Protein Data Bank (>35,000 sequences), the probability of a newly identified sequence having a structural homologue is actually quite high.

**Results:**

We have developed a method that performs structure-based sequence alignments as part of the secondary structure prediction process. By mapping the structure of a known homologue (sequence ID >25%) onto the query protein's sequence, it is possible to predict at least a portion of that query protein's secondary structure. By integrating this structural alignment approach with conventional (sequence-based) secondary structure methods and then combining it with a "jury-of-experts" system to generate a consensus result, it is possible to attain very high prediction accuracy. Using a sequence-unique test set of 1644 proteins from EVA, this new method achieves an average Q3 score of 81.3%. Extensive testing indicates this is approximately 4–5% better than any other method currently available. Assessments using non sequence-unique test sets (typical of those used in proteome annotation or structural genomics) indicate that this new method can achieve a Q3 score approaching 88%.

**Conclusion:**

By using both sequence and structure databases and by exploiting the latest techniques in machine learning it is possible to routinely predict protein secondary structure with an accuracy well above 80%. A program and web server, called PROTEUS, that performs these secondary structure predictions is accessible at . For high throughput or batch sequence analyses, the PROTEUS programs, databases (and server) can be downloaded and run locally.

## Background

The field of protein structure prediction began even before the first protein structures were actually solved [1]. Secondary structure prediction began [2,3] shortly after just a few protein coordinates were deposited into the Protein Data Bank [4]. In the 1980's, as the very first membrane proteins were being solved, membrane helix (and later membrane β-strand) and signal peptide prediction methods began to proliferate [5]. Homology modeling, as a way of predicting 3D structures, followed in the mid 1980's [6]. Later, in the 1990's the concept of threading (both 2D and 3D) emerged, thereby allowing reasonably accurate fold prediction to be performed on very distantly related sequences [7,8]. Over time, the accuracy and reliability of most protein structure prediction methods has grown considerably. This is, in part, due to the development of more sophisticated prediction methods using neural nets or Hidden Markov Models [9], the development of more rigorous testing methods [10,11] and the explosive growth in both sequence and structure data on which scientists can "train" their software (35,000+ structures and 2,000,000+ sequences).

Protein structure prediction continues to be an actively developing field with more than 400 papers being published on the subject each year. Incremental improvements in prediction accuracy are still being reported and until "the protein folding problem" is formally solved, it is likely that protein structure prediction will continue to be an active area of research and development [12]. The continuing improvements in structure prediction accuracy are also having an effect on how proteins are analyzed and annotated. While once an anathema to most protein chemists, secondary structure prediction is now becoming a routine part of many protein analyses and proteome annotation efforts [13]. Annotation systems such as PEDANT [16], BASYS [14], BACMAP [17], PSORTB [15] and others all depend on large scale secondary structure predictions to assist in identifying possible functions, to determine subcellular locations, to assess global trends in secondary structure content among different organisms or certain organelles, to identify protein folds or to enumerate fold classes (all alpha, all beta, mixed), to identify domains, and to estimate the proportion of "unfolded" or unstructured proteins in a given genome [20-22,27-29]. Likewise protein secondary structure predictions can play a valuable role for molecular biologists in deciding where and how to subclone protein fragments for expression (i.e. where to cut the gene), where to join or insert gene fragments, or in choosing where to add affinity tags for protein purification [26,27]. Secondary structure predictions can also be used to calibrate CD and FTIR measurements when monitoring the folding or unfolding proteins with no known 3D structure [19,18]. Secondary structure predictions may also be used to assist in the assignment of NMR spectra (of known or novel proteins), to re-reference chemical shifts and to help determine protein flexibility [23,24].

Currently the performance (or Q3 score) of the best secondary structure prediction methods, such as PSIPRED [35], JNET [36] and PHD [13,37] is between 75–77%. These methods, which are specific to water-soluble proteins, utilize BLAST or PSI-BLAST searches of the non-redundant protein sequence database to obtain evolutionary information. This information is then fed through a multi-layered feed-forward neural network that has previously been trained on known structures and known alignments to learn characteristic sequence/structure patterns. Those patterns are then used to predict the secondary structure of the query protein [38]. Similarly good scores can also be achieved using Hidden Markov Models with programs such as SAM-T02 [39]. More recently approaches that combine multiple high quality methods (a "jury of experts" or meta methods) have been described [40,41] and these appear to do even better than the single-pass prediction approaches.

What is somewhat surprising about the methods described so far is that they do not fully exploit the information that is available in the protein structure databases. So far as we are aware, none of the above-mentioned methods attempt to find sequence homologues in the PDB and to use the known secondary structure of those homologues to assign, map or predict the secondary structure of the query protein. As a rule, this sequence/structure alignment approach to secondary structure assignment is normally reserved for homology modeling programs [7,42]. For pairwise sequence identities of >35%, these secondary structure mappings are typically more than 90% accurate. However, we believe this 3D-to-2D mapping approach to general secondary structure prediction is not being fully exploited. A recent survey has found that less than 3% of new protein structures deposited into the PDB have a totally novel fold [43]. Even among structural genomics projects, where novel folds are explicitly being sought and solved, less than 10% of the targets exhibit completely novel folds [44,45]. Furthermore, we have found that nearly 3/4 of newly deposited PDB structures have sequence identities greater than 25% to a pre-existing structure. In other words, the vast majority of newly solved proteins could have at least a portion of their secondary structures predicted via this simple 3D-to-2D mapping approach. Thus, by combining a PDB-based structure alignment with a high quality *de novo *structure prediction program it may be possible to achieve a much higher overall Q3 score for protein secondary structure prediction.

Here we wish to describe a program, called PROTEUS, that exploits this concept of 3D-to-2D mapping and integrates it with multiple *de novo *methods to accurately predict protein secondary structure. Specifically, PROTEUS achieves an average Q3 score of 88% when tested on newly solved protein structures. This level of accuracy is 12–15% above that previously reported [35-41]. If a query protein has at least some portion of its sequence that is homologous to an existing PDB structure, the average Q3 score exceeds 90%. If absolutely no homology is found, or if the 3D-to-2D mapping option is turned off, the average accuracy of this method is still above 79%. In addition to greatly improving the average performance of secondary structure prediction, we have parallelized the prediction algorithm, developed a simple installation protocol and made the full source code and all associated databases freely available and as portable as possible. This was done in an effort to facilitate proteome annotation and to encourage large scale pipelined analyses or proteome-wide structure predictions to be done locally rather than remotely.

## Implementation

**Figure 1:**
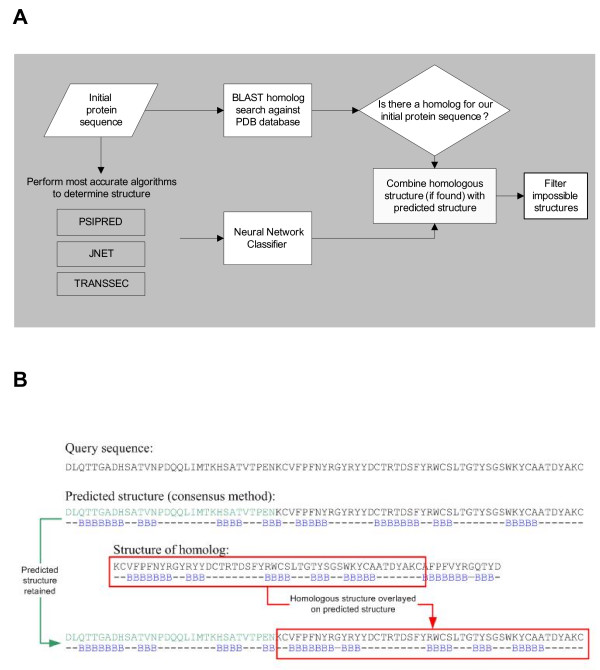
A) Flow chart outlining how protein sequences are processed by PROTEUS. Each query sequence is simultaneously processed through PROTEUS' three de novo predictors (lower left corner) and through a BLAST comparison and global alignment (via XALIGN) against the PDB, to yield a 3D-to-2D mapping. The two secondary structure predictions are merged and filtered to produce a final consensus prediction. B) Detail illustrating how the two predictions are merged in the final processing step.

PROTEUS consists of three components: 1) a large (12,464 entries), non-redundant and continuously updated database of sequences with known secondary structures; 2) a multiple sequence alignment algorithm for secondary structure mapping and homology prediction and 3) a "jury-of-experts" secondary structure prediction tool consisting of three different, high-performing de novo secondary structure prediction programs (PSIPRED, JNET and a home-made tool called TRANSSEC). The prediction algorithm itself involves four steps including an initial search against the PDB sequence database to determine if all or part of the query sequence is similar to a known structure. If a hit is found, a secondary structure mapping is performed on whatever component that mapped to the query. In the second step, a *de novo *secondary structure prediction using our three different (JNET, PSIPRED and TRANSSEC), high quality neural network (NN) approaches is performed. In the third step these three NN predictions are then fed as inputs into a fourth neural network, which then combines these predictions to make a prediction of its own (i.e. a decision by a jury of experts). Finally, the jury-of-experts prediction and the results of the initial homology search are combined to produce the final secondary prediction for PROTEUS (see Figure 1). Combining the two prediction methods allows PROTEUS to fill in any prediction gaps derived from the initial 3D-to-2D mapping process and always yields a full sequence prediction, regardless of the extent of sequence overlap to a PDB hit.

Key to the success of PROTEUS is its effective use of secondary structure databases. PROTEUS' secondary structure database (PROTEUS-2D) is assembled from a non-redundant version of the Protein Data Bank (PDB) in which all sequences with >95% sequence identity to any other sequence were removed using the CD-HIT utility [47]. Each sequence was then assigned a secondary structure using VADAR [48]. The secondary structures were then checked and filtered so as not to contain "impossible" structures, such as sheets or helices containing a single residue. VADAR uses a consensus method of identifying secondary structures that closely matches "simplified" DSSP [49] structure assignments (8 state to 3 state), STRIDE [50] and generally agrees well with manual secondary structure assignments made by X-ray crystallographers and NMR spectroscopists. In fact, using the PROTEUS-2D database of secondary structures, the performance of PSIPRED and JNET was actually found to improve slightly over the performance quoted for DSSP-assigned secondary structures (77% vs. 75%). The secondary structure content of the PROTEUS-2D database, which currently contains over 2.2 million residues from more than 12,400 sequences, is 33% helix, 29% beta sheet and 38% coil. Because of its critical importance to the prediction process, the entire PROTEUS-2D database is automatically updated on a weekly basis. This database is also freely available for download at the PROTEUS website.

The PDB homology search and 3D-to-2D mapping process in PROTEUS both employ BLAST (using the default BLOSUM 62 scoring matrix and standard gap penalty parameters) to score and align high scoring hits found in the PROTEUS-2D database. Those database sequences having an expect score greater than 10^-7 ^to the query sequence are retained for further analysis. This optimal expect value was determined by extensive testing with cut-offs ranging from 10^-1 ^to 10^-15^. Depending on the length and domain structure of the query sequence up to 20+ homologues may be identified by this process. The pairwise BLAST alignments are then used to assemble a multiple sequence alignment over the length of the query sequence. The resulting multi-sequence alignment is then used to directly map the secondary structure of the PROTEUS-2D database sequences (or a portion thereof) to the query sequence. The mapping process involves sliding a 7 residue window over each aligned sequence and assigning a similarity score (based on the sequence identity over that 7 residue window to the query sequence) to the central residue. The sequence with the highest "identity score" for any given residue is then privileged to assign its secondary structure to the aligned residue in the query sequence. In this way the secondary structure of the query sequence is essentially predicted by homology. For those query sequences that are predicted in this manner (with more than 95% sequence coverage), PROTEUS also produces an image of the approximate 3D fold using the PDB coordinates to generate the picture.

In situations where no homologue is found, or only a portion of the query sequence could be predicted by 3D-to-2D mapping (as might be found in multi-domain proteins), PROTEUS resorts to a jury-of-experts prediction to cover the unpredicted portion. This jury-of-experts approach uses three neural net predictors: PSIPRED [35], JNET [36] and our own TRANSSEC (Q3 = 70%, SOV = 73%) methods. The results from these predictors are then fed into a fourth neural network to produce a consensus prediction in a manner similar to that described previously [40].

The methods and underlying theory to PSIPRED and JNET have been published previously and the programs were used as received without further modification. The TRANSSEC program was developed in-house using a Java-based neural network package known as Joone [51]. TRANSSEC's underlying approach is relatively simple, consisting of a standard PSI-BLAST search integrated into a two-tiered neural network architecture. The first neural network operates only on the sequence, while the second operates on a 4 × N position-specific scoring matrix consisting of the secondary structure determined via the first network. The first neural net uses a window size of 19, and was trained on 1000 sequences from the PROTEUS-2D database (independent from those used in training the other neural nets). This neural net had a 399-160-20-4 architecture (21 × 19 inputs, 2 hidden layers of 160 and 20, and four outputs) and typically predicts the secondary structure of any given protein with a Q3 = 64–65%. TRANSSEC's neural net secondary structure predictions are performed on all PSI-BLAST homologues to the query sequence These homologues are then multiply aligned using XALIGN [46] with the secondary structure serving as a guide to place gaps and insertions. The resulting secondary structure-based alignment (and corresponding confidence scores) is then used as input for a second neural network. TRANSSEC differs from most other prediction programs (PHD, PSIPRED) in that the predicted secondary structure, instead of the sequence, is used as input for the second neural network. What TRANSSEC attempts to do is to learn, via a neural net, how to "average" aligned secondary structures in a more intelligent way. A simple averaging of secondary structures typically reduces the prediction accuracy from 65% (for a single prediction) to 63% (for the averaged prediction), while using a neural net increases the performance by about 7% over naive averaging. The second neural net in TRANSSEC was trained on 1000 sequences from the PROTEUS-2D database, and achieved a Q3 score of 70% and a SOV score of 72%. It used a window size of 9, and was based on a 36-44-4 architecture.

The jury-of-experts program, (JOE) which combined the results of the three stand-alone secondary structure predictions was also developed using Joone. JOE consisted of a standard feed-forward network containing a single hidden layer. Using a window size of 15, the structure annotations and confidence scores from each of the three methods (JNET, PSIPRED, and TRANSSEC) were used as input. The JSP neural net was trained and tested (using a leave-one-out approach) on 100 sequences chosen randomly from the non-redundant database mentioned above. Four output nodes were used, one for each of helix, strand or coil, as well as a fourth denoting the beginning and end of the sequence. A back-propagation training procedure was applied to optimize the network weights. A momentum term of 0.2 and a learning rate of 0.3 were used, and a second test set of 20 proteins was applied at the end of each epoch, to ensure that the network was trained for the most optimal number of iterations. The JOE program outputs not only the secondary state call (H for helix, C for coil and E for beta strand), but also a numeric confidence score (ranging from 0 to 9, with 9 being most confident). Relative to simple averaging, the JOE program is able to improve secondary structure predictions by an average of 3% (79.1% vs. 76.4%). The improvement achieved using this jury of experts approach is likely due to the fact that JNET, PSIPRED and TRANSSEC perform differently for different types of proteins, with one method typically outperforming the other two depending on the secondary structure content, protein length or amino acid content. It appears that JOE's neural net was able to learn which method or which segmental prediction to trust more and therefore to place more weight on those predictions. It also appears that the JOE method also learned to modify the JNET and PSIPRED predictions (typically by lengthening them) to conform better to the VADAR-assigned secondary structures.

The final step in the PROTEUS algorithm involves merging the homology prediction (if available) with the jury-of-experts predictions. The PROTEUS-merge program was designed to accommodate three situations: 1) the case where no PDB homologue could be found, 2) the case where complete 3D-to-2D mapping was achieved and 3) the case where the 3D-to-2D mapping provided only partial coverage of the full query sequence. In the simple situation where no 2D-to-3D prediction is available (Case 1), the merge process simply takes the jury-of-experts or *de novo *result. Similarly, if a complete PDB-based secondary structure prediction is available (Case 2), the jury-of-experts prediction is generally ignored. In particular, if the homologue confidence score is equal to or greater than the consensus *de novo *score, then the homologue structure assignment is retained. Otherwise the *de novo *structure assignment is kept. Typically the *de novo *confidence scores range from 3–9, while the homologue confidence scores range from 8–9. The confidence of a homologue prediction is based on the running average (over a 7 residue window) of the sequence identity between the query sequence and that of the top matching PDB homolog. If the sequence identity is less than 30% (or 2/7), the confidence score assigned to the middle residue in the window is 8. If it is greater, the confidence score of the middle residue is 9. Confidence scores for the consensus *de novo *predictions are determined by the weightings of specific neural network nodes. If a homologous sequence or a group of homologous sequences is found (as with multi-domain proteins) that did not cover the entire length of the query sequence (Case 3), the unpredicted or unmapped portion is assigned the secondary structure determined by our Jury-of-experts approach (Figure 1).

PROTEUS also has a number of I/O utilities and interface tools that allow it to accept protein sequences (in FASTA and Raw format) and to produce colorful and informative output including all sequence alignments, corresponding BLAST scores, sequence matches, confidence scores, colored secondary structure annotation as well as 3D images of any modeled structures. Additional data handling and task handling tools were also written to manage the server side of the program, to update the PROTEUS-2D database on a weekly basis, and to parcel out tasks to other processors in a parallel fashion. The programs used to create PROTEUS and the PROTEUS web server were written in both C and Java 1.5. Specifically, XALIGN, VADAR, JNET, BLAST and PSIPRED were written in ANSI standard C, while TRANSSEC, the Jury-Selector, most of the input/output handling routines, as well as the web server interface were written in Java. The PROTEUS-2D update script was written in Perl.

## Results

PROTEUS' performance was tested in four different ways, 1) through leave-one-out testing on a set of 100 training proteins from the PROTEUS-2D database; 2) through a "blind" test and comparison on the latest EVA training set (1644 proteins); 3) through analysis of 125 randomly chosen proteins that were recently solved by X-ray and NMR; and 4) through direct comparisons of 10 randomly chosen proteins to well-known secondary structure web servers. The intent of these different tests was to gain some understanding of the performance of PROTEUS under different prediction situations and to assess its performance relative to other well known predictors. For the first test, the performance of the jury-of-experts system was assessed using a leave-one-out strategy on 100 randomly chosen proteins form the PROTEUS-2D databases. As previously mentioned, this method achieved a Q3 score of 79.1% and a SOV score of 77.5%. When this method was combined with the 3D-to-2D mapping (excluding identical matches from the PROTEUS-2D database), the performance was Q3 = 88.0% and SOV = 86.5%. The performance for the "full" version of PROTEUS (*de novo *plus homologue mapping) is about 10–15% higher than previously reported for other methods. Because this first test was done on training data (albeit using a leave-one-out strategy) it might be argued that the high performance may be due to overtraining or to the small sample size.

**Figure 2:**
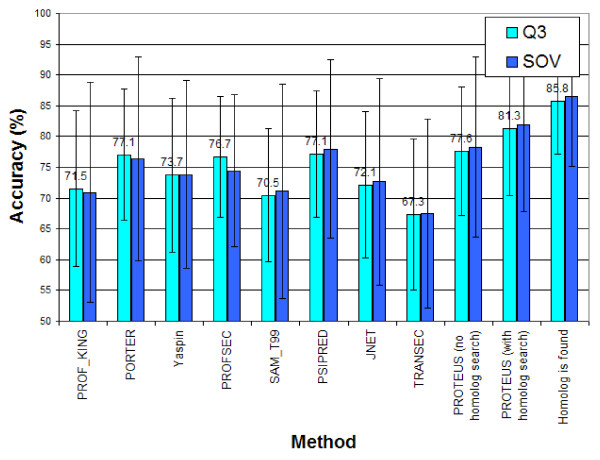
Hisotgram comparing the Q3 (light) and SOV (dark) scores of PROTEUS (right 3 bars) versus PSIPRED, JNET, and TRANSSEC for the test set (N = 1644) of non-homologous EVA sequences. Data for YASPIN, PORTER, PROF-KING, PROFSEC, and SAM-T99 are also shown. These were calculated from a smaller (N = 30–39) subset of sequences and predictions posted on the EVA website and on the PROTEUS home page. The Q3 score is written at the top of each predictor's set of bars. Standard deviations are shown as error bars.

To more legitimately assess the performance of PROTEUS a second "blind" test was done on data not part of PROTEUS' training set and for which no PDB homologues would be expected. Specifically the most recent release (March 2006) of the EVA [11] sequence-unique subset of the PDB was downloaded and used to measure the performance of PROTEUS. The EVA collection represent a set of non-homologous proteins that do not match any 100+ residue segment of any other protein in the PDB with greater than 33% sequence identity. The EVA test-set has been used for a number of years to benchmark protein secondary structure predictors, particular for CASP competitions [11]. The use of a sequence-unique data set such as EVA is intended to simulate the situation where one might be predicting secondary structures in a structural genomics project, where novel fold identification is key. In this particular situation one would expect that the PROTEUS predictions would be dominated by its *de novo *methods and that the Q3 and SOV scores would be somewhat reduced over the first test. A total of 1644 protein sequences and PDB ID's were obtained from the EVA website and the secondary structure for each of the test-set proteins was assigned by VADAR [48]. PROTEUS was then used to predict the secondary structures and the performance was evaluated against the VADAR-assigned secondary structures. The program was tested in two modes, one with the PDB homologue search turned off (*de novo *prediction only) and other with the PDB search turned on. In both cases the Q3 and SOV scores were calculated for each protein in the 1600 protein test set. Note that the SOV score is similar to Q3 but more sensitive to the segment grouping or overlap of secondary structure elements [52]. At the same time the Q3 and SOV scores for JNET (alone) and PSIPRED (alone) were also determined for all 1644 EVA proteins. Additionally the secondary structure predictions posted on the EVA server for PORTER [31], PROF-KING [32], PROFSEC [34], SAM-T99-sec [33] and VASPIN [30] were also downloaded and processed in a similar manner to the PSIPRED and JNET predictions. Note that the number of predictions for these predictors was much less than 1644 as the EVA server often only performs a small number (<200) of predictions for any given predictor. As seen in Figure 2, PROTEUS achieves a Q3 of 77.6% (SOV = 78.2%) when its homologue search is turned off and a Q3 score of 81.3% (SOV = 81.8%) when the homologue search is turned on (with the exact match in the PDB removed), with a standard deviation of 11.0% and 14.1%, respectively. Evidently, even in a sequence-unique data set, some fragmentary homology is still detectable by PROTEUS. In particular for those proteins that exhibited some detectable homology to a portion of a PDB structure, the performance was actually quite good (Q3 = 85.8%, SOV = 86.5%). Comparisons to other predictors on the same set or proteins (PSIPRED, JNET) or a subset of these proteins (PORTER, PROF-KING, etc.) indicate that these methods perform at levels from 70.5%–77.1% (Q3) or 70.9%–77.9% (SOV). The Q3 and SOV scores we obtained for these predictors on our EVA test set are very close (<1% difference) to those reported by the authors or posted on the EVA website. While the performance of PROTEUS is not quite as impressive as seen in the first test, it still demonstrates that under strict "CASP" testing conditions, PROTEUS performs approximately 4–8% better than other high-performing secondary structure predictors.

**Figure 3:**
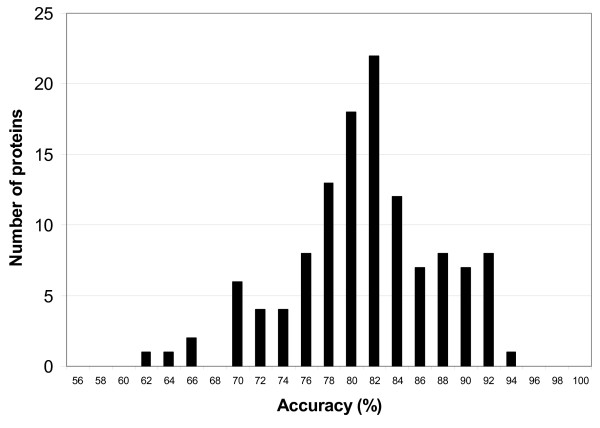
Histogram illustrating the distribution of accuracy (Q3) scores (%) for PROTEUS' de novo secondary structure predictions (i.e. with the PDB homologue search turned off) as measured on the complete test set of 125 PDB entries. The mean is 79.7% and the standard deviation is 7.5%.

**Figure 4:**
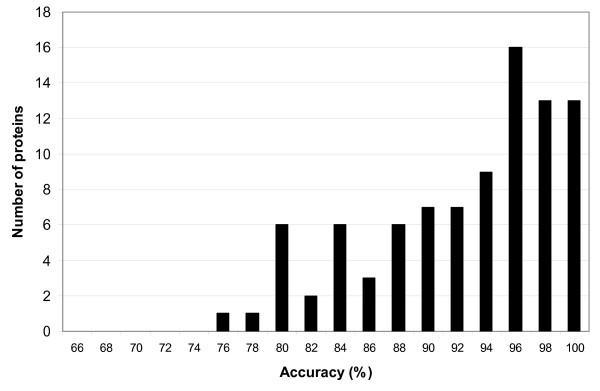
Histogram illustrating the distribution of accuracy (Q3) scores (%) as measured on the test set of 88 proteins that had homologs (E > 10^-7^) to existing PDB entries. The mean is 90.0% and the standard deviation is 6.3%.

**Figure 5:**
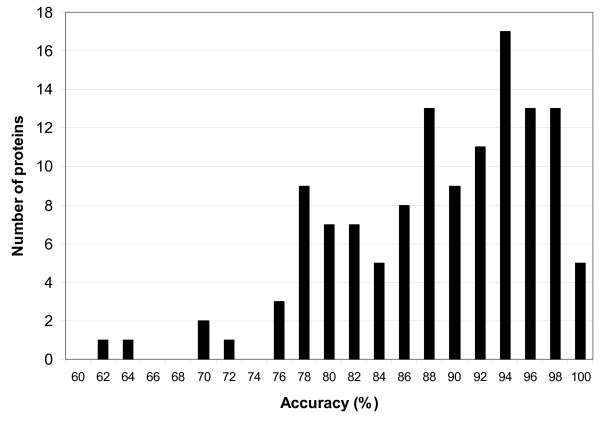
Histogram illustrating the distribution of accuracy (Q3) scores (%) of the consensus prediction from PROTEUS as measured on the test set of 125 proteins. The mean is 87.8% and the standard deviation is 10.2%.

**Figure 6:**
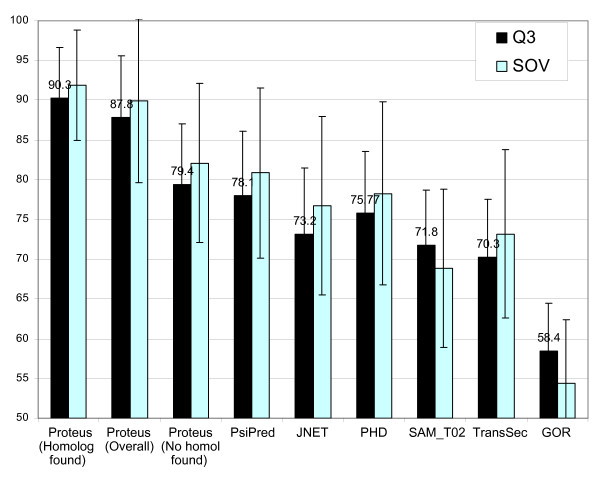
Hisotgram comparing the Q3 (black) and SOV (gray) scores of PROTEUS (left 3 bars) versus PSIPRED, JNET, PHD, SAM-T02 and GOR for test set of 125 proteins. The Q3 score is written at the top of each predictor's set of bars. Standard deviations are shown as error bars.

The third test of PROTEUS' performance was intended to simulate the situation where one is trying the predict the secondary structure of proteins that are being studied by X-ray and NMR, but not yet solved, not yet published or not yet released by the PDB. This kind of test is intended to answer the question: What is the secondary structure prediction performance of PROTEUS for proteins that are of interest to genome annotators, structural biologists or protein chemists? A testing set of 125 randomly chosen, non-redundant, water soluble proteins was generated by downloading the PDB coordinates of a subset of proteins deposited from January 2005 to June 2005. Because the training set of proteins originally used to refine and optimize PROTEUS consisted of proteins deposited into the PDB prior to December 2004, this precluded any possibility of testing on the training set. As with the previous tests, the secondary structure for each of the test-set proteins was assigned by VADAR [48]. PROTEUS was then used to predict the secondary structures (with the homologue search turned on or off) and the performance was evaluated against the VADAR-assigned secondary structures. Figure 3 summarizes the distribution of Q3 scores for PROTEUS as tested over the entire 125 protein test suite, with the homologue search turned off (i.e. using the *de novo *prediction only). The average score in this case was 79.7% (Q3) and 82.0% (SOV) with a standard deviation of 7.5% and 10.3%, respectively. Figure 4 displays the distribution of PROTEUS' Q3 scores with the homologue search turned as applied to the 88 proteins in the test set for which a PDB homologue (with an expect score >10^-7^) was found. In other words, 70.4% of the test proteins could have their secondary structure predicted via 3D-to-2D mapping. The average score for the 88 homologues was 90.0% (Q3) and 91.8% (SOV) with a standard deviation of 6.3% and 7.0% for Q3 and SOV scores respectively. Therefore PROTEUS' combined, consensus prediction (Figure 5) for all 125 test proteins yielded an average accuracy of 87.8% and 90.0% for Q3 and SOV scores respectively, with a standard deviation of 7.9% (Q3) and 8.7% (SOV). The low scoring outlier proteins (Q3 scores between 62%–70%) are typically very short peptides or proteins which have absolutely no homologue in the PDB. For further comparison the same test set (125 proteins) and testing procedures were used to evaluate the performance of several other high-performing secondary structure prediction methods including PSIPRED [35], JNET [36], SAM_T02 [39], as well as a locally written version of GOR [53] and our own TRANSEC. To ensure complete consistency, the BLAST database searches, which were required for all programs (except GOR), were performed on the same local copy of the non-redundant (NR) NCBI protein database. Figure 6 presents the results of these prediction programs in comparison to the predictions obtained with PROTEUS. A quick visual comparison reveals that PROTEUS' performance is significantly better (10–30%) than all five tested programs. For instance, PSIPRED, which is generally regarded as being one of the most accurate methods [11,35], obtained scores of 78.1% (Q3) and 80.9% (SOV) respectively. In comparison, PROTEUS' consensus method obtained scores of 87.8% (Q3) and 90.0% (SOV). Therefore, in this test, PROTEUS' scores were approximately 10% higher than those achieved by PSIPRED. Even when PROTEUS is partially disabled (the PDB homologue search is turned off) it still performs about 2% better than the best-performing routine (79.7% vs. 78.1%). The statistical significance of this 2% improvement was verified using a standard paired two-sample *t*-test, which confirmed that the two means were statistically different (p = 4.63 × 10^-7^, t-stat = 5.166, critical value = 1.657 with 124 degrees of freedom).

To verify that the performance differences noted in Figure 5 were not the result of improper program installation, limited tool selection or outdated software, we conducted a fourth test on a set of 10 recently (Sept, 2005) solved proteins using a number of popular secondary structure prediction web servers. Note that these 10 proteins were not contained in the PROTEUS-2D database. The proteins ranged in size from 76–502 residues. The results are summarized in Table 1. Once again, the results largely confirm what was seen in Figure 5, with PROTEUS averaging close to 90% in both Q3 and SOV and the others ranging between 55% and 75%. The performance of these servers in this test set is also consistent with what has been described in the literature [11,35-39]. Overall, these four independent tests confirm that PROTEUS is able to predict secondary structure of soluble proteins with very high accuracy. When restricted to the prediction of sequence-unique proteins (such as those found in EVA or those targets selected for structural genomics projects) PROTEUS has a Q3 of 81.3%, which as about 4–8% better than the best performing methods. When allowed to predict the structure of any generic protein (as might be done for a genome annotation project) PROTEUS has a Q3 of 88%–90% which is about 12–15% better than the best performing methods described to date.

## Discussion

PROTEUS was primarily developed to facilitate secondary structure prediction for genome annotation. In genome annotation one is primarily interested in getting the most correct annotations or the most accurate predictions in the quickest possible way. Making use of prior information or fragmentary data to fill in knowledge gaps is perfectly reasonable and strongly encouraged [16,14,21,22,29]. Likewise making this process as automated and fool-proof as possible is a basic requirement of genome annotation systems. If one is interested in getting the most complete and accurate secondary structure assignment of as many proteins as possible, then it is quite natural to want to combine an *ab initio *or *de novo *prediction method with a method that extracts known or partially known secondary structure assignments (from PDB data, from NMR NOE data, from MS/MS hydrogen exchange data) and to have this done automatically.

Perhaps the best way to appreciate the general utility of PROTEUS is to imagine a scenario where one is given the sequence of a large 840 residue protein (lets call it Vav1) and then asked to generate the most accurate or most correct secondary structure assignment for this protein. Suppose a BLAST search or CDD search reveals that this protein has 7 different domains, 4 of which have PDB homologues (2 of which have less than 35% sequence identity to a PDB target) and 3 other domains which have no known structure. To generate the most accurate possible secondary structure assignment for this multidomain protein would require many manual steps and a good deal of bioinformatics skill including: 1) a BLAST search against the PDB; 2) manual selection of the highest scoring homologues; 3) homology modeling using Swiss-Model [42] or another modeling server for the two homologous domains with >35% sequence identity; 4) assignment of the secondary structure for two of the domains using DSSP, STRIDE or VADAR; 5) sequence-based threading on the 3D-PSSM server [28] to generate possible folds of the remaining two low-scoring homologues; 6) manual assessment and adjustment of the predicted folds and their alignments; 7) prediction of the secondary structure of the remaining 3 domains using a *de novo *predictor such as PSIPRED or PHD and 8) manually typing, cutting or pasting all the secondary structure assignments on to every residue in the 840 residue sequence. A skilled bioinformatician might be able to do this in a couple of hours, an unskilled individual might take several days. Alternately, one may elect the easy route and simply predict the structure of the entire protein using a *de novo *structure predictor such as PSIPRED or PHD. However, choosing to do this would likely reduce the accuracy of the prediction by 10–15% (i.e. going from a Q3 of 85% to 75%).

Now suppose that one was asked to do this kind of high-end structure prediction not for just one protein but for 23,000 proteins (i.e. genome annotation) or that it has to be done on 4000 proteins every 2 weeks (the current rate at which new microbial genomes are being released). Clearly such a manual intensive process would have to be replaced by an automated technique. This is the primary motivation behind PROTEUS. PROTEUS effectively replaces 8 manually tedious steps with a single automated process. In fact, this 8 step example of Vav1 is not entirely hypothetical. The single step PROTEUS result (which takes about 2 minutes) for Vav1 is shown in the Sample Output on the PROTEUS homepage. Inspection of the output clearly demonstrates how PROTEUS can combine prior knowledge (PDB data) with *de novo *predictions to generate optimally accurate secondary structure assignments for large and complex proteins.

PROTEUS is able to achieve its very high level of accuracy because it brings together two high performing methods of secondary structure prediction – a novel *de novo *method based on a jury-of-experts approach and a novel 3D-to-2D homology mapping method. The 3D-to-2D mapping process is not completely unknown. In fact, it is frequently used as an intermediate step in several homology modeling programs to identify conserved structural scaffolds [7,42]. Given the well known fact that secondary structure is more conserved than primary structure, it stands to reason that mapping the secondary structure onto a given query sequence – even for remotely related homologues – will yield a high quality secondary structure "prediction". This is borne out by the fact that our mapping method is able to predict secondary structure with greater than 90% accuracy. This mapping approach is obviously limited to query proteins that have a homologue or potential homologue already deposited in the PDB database. As might be expected, the accuracy of the mapping prediction is generally tied to the level of sequence identity or BLAST expect value. Highly similar sequences (>80% identity) can have their secondary structure predicted with close to 90% accuracy. Intermediate similarity (40–80% identity) yields predictions that are 80–90% correct while low sequence identity (25–40%) yields secondary structure predictions that are 75–80% correct. This partly explains the distribution of scores seen in Figure 4.

Certainly, when the PDB was relatively small (prior to the year 2000), this 3D-to-2D mapping method would prove to be relatively ineffective. However, with the rapid expansion of the PDB over the past 5 years we are now able to take advantage of the fact that an increasingly large fraction of protein structures that are being solved or for which people want to know the structure, have at least one homologue in the Protein Data Bank. Indeed, less than 3% of all newly deposited structures have novel folds (and therefore novel secondary structure arrangements) and it appears that less than 10% of structural genomics targets are yielding truly novel folds [43-45]. Therefore, the odds that any given protein will have a novel arrangement or a unique order of secondary structures (which would reduce the accuracy of this homologue approach) is becoming relatively small. Even with the modest approach employed here (requiring sequence identity >25% or an E < 10^-7^), we still find that 70% of "testable" proteins have at least one homologue or a portion of a homologue in the PDB. Therefore, on average, the 3D-to-2D mapping process is going to be effective for about 70% of all query proteins which are solvable by today's X-ray and NMR methods. We would predict that this fraction (70%) would continue to increase as the PDB continues to expand and the number of known folds grows.

Note that this figure of 70% is not applicable if were to try to predict secondary structure for entire genomes. Large scale homology modeling efforts suggest that only about 30–50% of a given genome is amenable to homology modeling or threading [54]. Therefore if we applied the lower figure of 30% (for the probability of finding a PDB homologue in a newly sequenced genome) to our protocol we would predict the performance of PROTEUS in predicting the secondary structure of soluble proteins would drop to 83%. Note that this figure is still 7–10% better than existing secondary structure prediction methods. Obviously if one biased their selection of query proteins such that no portion of the sequence had any sequence homology whatsoever to something in the PDB, then PROTEUS could do no better than its *de novo *approach (about 78–79%), even with its PDB search turned on. Similarly, we would predict that genomes from poorly sampled branches of the tree-of-life would probably be less well predicted than those belonging to the better studied branches (mouse, yeast, humans, E. coli).

Given the potential variability in PROTEUS' predictions, we believed it was important to provide a reliability or confidence score in PROTEUS' prediction output. These reliability scores are determined on the basis of the neural network outputs (for the *de novo *predictions) or the level of sequence identity to a given PDB match (for the 3D-to-2D mapping method). Reliability scores are generated not only for each residue for each prediction, but also for each residue in the consensus (i.e. final) prediction and for the entire protein. The maximum reliability score is 9 (for a residue) and the maximum reliability score for a complete protein is 90%.

While PROTEUS' 3D-to-2D mapping procedure offers a number of advantages in secondary structure prediction, it is also important to remember that another key strength in PROTEUS lies in its *de novo *structure prediction routine. This jury-of-experts approach, which uses machine learning methods to combine three independent and high performing structure prediction algorithms into one, is able to consistently predict secondary structures with an accuracy approaching 79%. This is still 2% higher than any other single pass method with which we could directly compare. This consensus method uses PSIPRED, which generates BLAST sequence profiles to extract evolutionary and sequence information using a neural network; JNET, which uses a combination of solubility information, evolutionary information, and a Hidden-Markov Model/neural network combination; and TRANSSEC, a locally developed algorithm which uses a two-tiered prediction system to extract evolutionary similarities. These three methods are sufficiently "orthogonal" in their prediction methodology that the combination of the three is able to generate a consensus prediction that is 2–5% higher than any individual prediction. The ability to generate *de novo *secondary structure predictions which are consistently near 80% correct, especially in regions where the 3D-to-2D mapping approach fails, certainly helps to create consensus predictions that are consistently close to 88% correct.

While PROTEUS clearly performs very well, there are still a number of improvements or additions that could be made to the program. One obvious improvement could be the integration of conventional membrane spanning prediction routines and signal recognition programs [55] to make PROTEUS capable of handling all protein types (water-soluble, targeted and transmembrane proteins). This would be particularly useful in whole genome annotation applications. Another improvement could be made in PROTEUS' sensitivity in its 3D-to-2D mapping steps. By simply employing PSI-BLAST [56] instead of BLAST it should be possible to increase the fraction of PDB homologues (from 70% to ~80%) that could pass through the 3D-to-2D mapping steps. However, given the drop in predictive performance seen for homologues with <30% identity, it is not clear whether this would lead to a very substantial improvement in overall accuracy. Yet another potential addition to PROTEUS would be a 2D threading or fold prediction service. Given the high accuracy of its secondary structure predictions, one might expect that PROTEUS could yield somewhat more reliable results and somewhat improved fold classifications.

Along with its high accuracy and its ready availability as a web server, we have also ensured that the downloadable version of PROTEUS would be a well-documented, user-friendly system which is easy to install and does not require additional input or obscure pre-processing steps. During our testing processes we found that many other systems offered relatively limited documentation, required the user to provide additional inputs, such as an alignment and BLAST output files, or demanded that additional scripts or programs to be run to compile the input into a suitable format. Often users will not know how to supply these extra inputs (for example, creating a list of aligned sequences in a special format). Given these difficulties, we have tried to make the installation and operation of PROTEUS as simple as possible. The local version of PROTEUS (see Availability and Requirements section) requires nothing more than a sequence in either FASTA or Raw format. The output can be customized, and due to its open source nature, modular design and extensively commented Java code, the algorithms can be incorporated easily into other applications for batch or online processing. PROTEUS was also designed to take full advantage of multi-processor systems and should scale well as computational resources increase. This is a particularly important consideration in genome/proteome annotation efforts.

PROTEUS' software does have a few drawbacks. Because it is written in Java, it requires substantial memory to run. Furthermore, the neural networks used in the program were not optimized for minimal memory use; therefore PROTEUS requires at least 512 MB of RAM to be allocated to the Java Virtual Machine. With increasing hardware availability and lower prices, this requirement should not be too much of a concern in the future. Additionally, because of the requirement to run three independent *de novo *prediction methods, a 3D-to-2D mapping step and a consensus prediction generator, PROTEUS is somewhat slower than other methods. While PSIPRED can typically return a result within seconds of completing a lengthy PSI-BLAST search, PROTEUS requires almost a minute to complete its predictions (in addition to a PSIBLAST search). Efforts are being made to reduce this time requirement with further code optimization and multi-processor utilization.

## Conclusion

PROTEUS is both an integrated web server and a stand-alone application that exploits recent advancements in data mining and machine learning to perform very accurate protein secondary structure predictions. PROTEUS combines three high-performing *de novo *structure prediction methods (PSIPRED, JNET and TRANSSEC), a jury-of-experts consensus tool and a robust PDB-based structure alignment process to generate all of its secondary structure predictions. For water-soluble protein PROTEUS is able to achieve a very high level of accuracy (Q3 = 88%, SOV = 90%) which is approximately 12–15% higher than that previously reported [35-41]. The program's performance was extensively tested and compared to both available programs and publicly accessible web servers using a variety of test proteins and test scenarios. In all cases PROTEUS appears to perform better than existing tools. This performance improvement is statistically significant and robust. In the rare situations (20–30%) where a query protein shows no similarity whatsoever to any known structure, or if the 3D-to-2D mapping option is turned off, PROTEUS is still able to achieve a Q3 score of ~79%. This is still statistically better than what has been reported elsewhere. However, it is still important to be somewhat circumspect in interpreting these results. The standard deviation for essentially all secondary structure prediction routines (including PROTEUS) still stands at ~10% and so some caution must be exercised in interpreting or relying upon these predictions. Indeed, it is theoretically possible to get a PROTEUS prediction that is only 50% correct. Until a method is developed where the standard deviation in prediction accuracy is <5% or until the PDB expands to encompass all "fold space", there is still a strong need to develop better routines and more complete databases. To facilitate further algorithmic improvements, widespread adoption, and easy incorporation into genome annotation pipelines, PROTEUS was designed to be completely open source. Given its high accuracy and open-source nature, we believe PROTEUS could make a very useful addition to the current arsenal of structure prediction tools available to protein chemists, genome annotators and bioinformaticians.

## Availability and requirements

The PROTEUS website is accessible at . The entire PROTEUS suite occupies approximately 1.2 GBytes of data with the PROTEUS-2D database occupying 5.2 Mbytes and the NR protein sequence database occupying 1.1 Gbytes. All programs were tested and compiled on a variety of UNIX platforms and should work on most systems operating Linux and Mac OSX (10.4+). All programs and databases are downloadable at  and are supported with an easy-to-use installation script. A typical PROTEUS run for a 300 residue sequence takes approximately 3 minutes on a 2.8 GHz machine equipped with 1 GB of RAM.

## Authors' contributions

SM wrote, tested and installed most of the predictive software described here, designed and conducted all performance tests and prepared the first draft of the manuscript, SS wrote and tested the software used to generate the PROTEUS-2D database, WJG provided direction, ideas and critical suggestions in the early phases of the project, DSW wrote the final manuscript, conceived of the central ideas in the paper and coordinated most of the project.

## Figures and Tables

**Table 1:** Secondary structure prediction accuracy of PROTEUS versus that obtained with 7 different protein prediction servers using an identical set of 10 recently solved non-membrane proteins. Note that the PHD server was not included since it was used in the 125 protein test shown in Figure 6.

Method	Proteus	HNN [56]	JPRED [57]	PSIPred [58]	SSPro4 [59]	Porter [60]	Sopma [61]	Prof [62]
Q3	89.9	58.1	74.2	75.9	75.7	75.6	62.3	74.2
SOV	90.3	58.8	77.9	76.1	74.9	75.6	62.3	77.8
